# Bioplating demonstrates increasing micromovement during cyclic loading after anterior closed wedge high tibial osteotomy despite no significant difference in failure load compared to angle‐stable plate fixation

**DOI:** 10.1002/jeo2.70911

**Published:** 2026-09-11

**Authors:** Andrzej Jasina, Pia Metje, Jan‐Oliver Sass, Jessica Hembus, Sjard Simons, Sina Gräber, Kira Liebau, Rainer Bader, Philipp Schuster, Maeruan Kebbach, Parisa Pourostad, Christoph Lutter, Felix Ferner

**Affiliations:** ^1^ Department of Orthopaedics Rostock University Medical Center Rostock Germany; ^2^ Centre for Sports Orthopedics and Special Joint Surgery Orthopedic Hospital Markgroeningen Markgröningen Germany; ^3^ Department of Orthopedics and Traumatology, Clinic Nuremberg Paracelsus Medical University Nuremberg Germany; ^4^ Department of Orthopaedic Surgery Hospital Lichtenfels Lichtenfels Germany

**Keywords:** ACL revision, biomechanics, knee joint, osteotomy, posterior tibial slope

## Abstract

**Purpose:**

To biomechanically compare fixation stability after anterior closed wedge high tibial osteotomy (ACW‐HTO) for posterior tibial slope (PTS) correction using trans‐tuberosity “bioplating” and infra‐tuberosity angle‐stable plate fixation.

**Methods:**

In this cadaveric biomechanical study, fresh‐frozen human knee specimens were allocated in a paired donor‐matched manner to the two intervention groups. Tensile load was applied to the quadriceps tendon at a fixed flexion angle using an electrodynamic testing machine. After preloading, 1000 load cycles between 50 N and 200 N were performed at 1 Hz. The specimens were then loaded to failure at 10 mm/s. Micromovement at the osteotomy gap was analysed after 200 and 800 cycles using an optical 3D system. Two‐way ANOVA with Bonferroni multiple comparison testing was used, with *p* < 0.05 considered statistically significant.

**Results:**

Twelve cadaveric knee specimens (*n* = 6 per intervention group; mean age 79.2 ± 8.2 years) were included. No statistically significant difference in micromovement was found between the two intervention groups after 200 (*p* = 0.515) or 800 cycles (*p* = 0.889). In the “bioplating” group, micromovement increased from 0.055 ± 0.017 mm after 200 cycles to 0.069 ± 0.035 mm after 800 cycles (*p* = 0.025), whereas no significant increase occurred with angle‐stable fixation (*p* = 0.518). Osteosynthesis failure load did not differ significantly between groups (643.9 ± 190.8 N vs. 566.8 ± 111.0 N, *p* = 0.422).

**Conclusion:**

No significant difference in failure load was detected between the techniques. However, “bioplating” demonstrated increasing micromovement during cyclic loading. Considering the limitations of the present biomechanical setup, superiority or inferiority of either fixation technique cannot be concluded. These findings should serve as a basis for further biomechanical testing and should be considered when selecting the surgical technique and postoperative treatment regimen in patients undergoing ACW‐HTO for PTS correction.

**Level of Evidence:**

Level V, controlled laboratory study.

## Graphical abstract

One‐sentence summary of the main finding: Bioplating showed increasing micromovement during cyclic loading after ACW‐HTO, while failure load did not differ significantly from angle‐stable plate fixation.

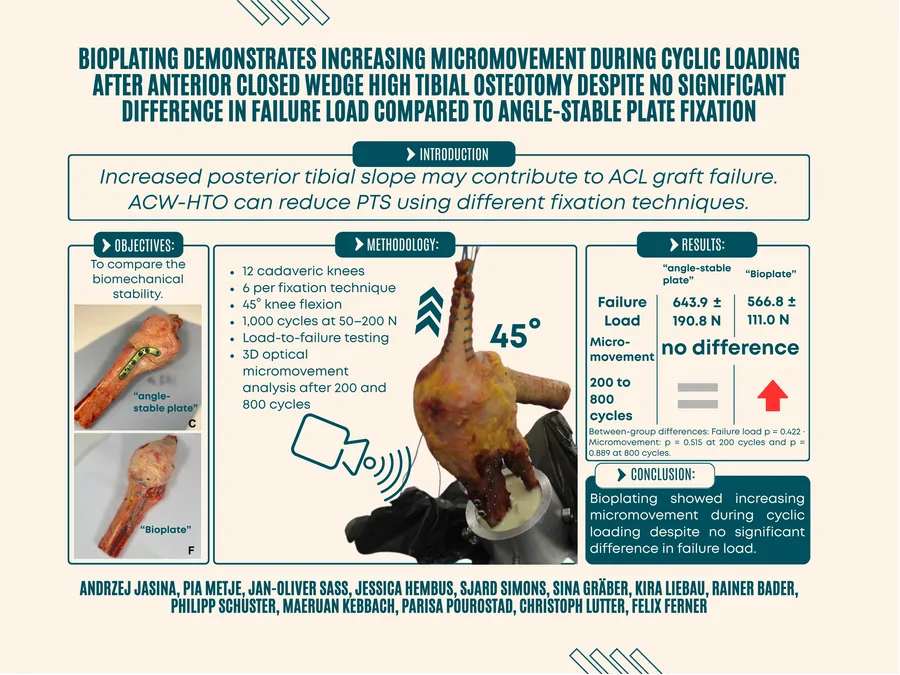

**Abbreviations**
ACL: anterior cruciate ligament
ACW‐HTO: anterior closed wedge high tibial osteotomy
ANOVA: analysis of variance
BMD: bone mineral density
DXA: dual‐energy X‐ray absorptiometry
Hz: Hertz
mm: millimetres
mm/s: millimetres per second
N: Newtons
PCL: posterior cruciate ligament
PTS: posterior tibial slope
ROM: range of motion
SD: standard deviation

## INTRODUCTION

It is well known that an increased posterior tibial slope (PTS) increases the forces on the anterior cruciate ligament (ACL) [1, 3, 6, 11, 20, 23, 31]. This has strongly influenced ACL revision surgery methods over the last few years, since, among other factors such as tunnel position, graft selection, and meniscal injuries, increased PTS also appears to be a relevant risk factor for graft failure in ACL revision surgery [12, 13, 14, 18, 26, 28, 29].

Hence, different surgical techniques have been established in order to reduce pathologically increased PTS [7]. Due to the anatomical approach to the tibial head, anterior closed wedge high tibial osteotomies (ACW‐HTO) have been most commonly implemented when correction in the sagittal plane alone was intended [30]. Three techniques were developed, which differ in their relation to the tibial tubercle: (1) Osteotomy proximal to the tibial tubercle [4]. (2) Osteotomy trans‐tuberosity with detachment of the tibial tubercle [2, 25]. (3) Osteotomy distal to the tibial tubercle [9]. Whereas the first technique with the osteotomy proximal to the tibial tubercle (“retrotubercle”) was considered stable due to the compression of the patellar tendon forces on the osteotomy site, the other two techniques were considered less stable, which has not yet been demonstrated biomechanically.

Moreover, one of the main differences between these two surgical approaches is the technique of osteosynthesis: In the trans‐tuberosity technique the detached tibial tubercle is used as a “bioplate” and fixation is achieved by three to eight lag screws, whereas with the infra‐tuberosity technique the fixation is achieved by an angle‐stable plate with compression on the osteotomy. A stable osteosynthesis is important to enable early full weight‐bearing and full passive and active range of motion of the knee joint in these young and active patients. To date, there is no evidence about the biomechanical aspects of these two fixation techniques.

The primary aim was to compare load‐to‐failure between both fixation techniques, hypothesising no significant difference between groups. The secondary aim was to compare osteotomy‐gap micromovement after 200 and 800 loading cycles, hypothesising no significant between‐group difference or cycle‐dependent increase.

## MATERIAL AND METHODS

### Preparation of specimens and surgical technique

**Figure 1:**
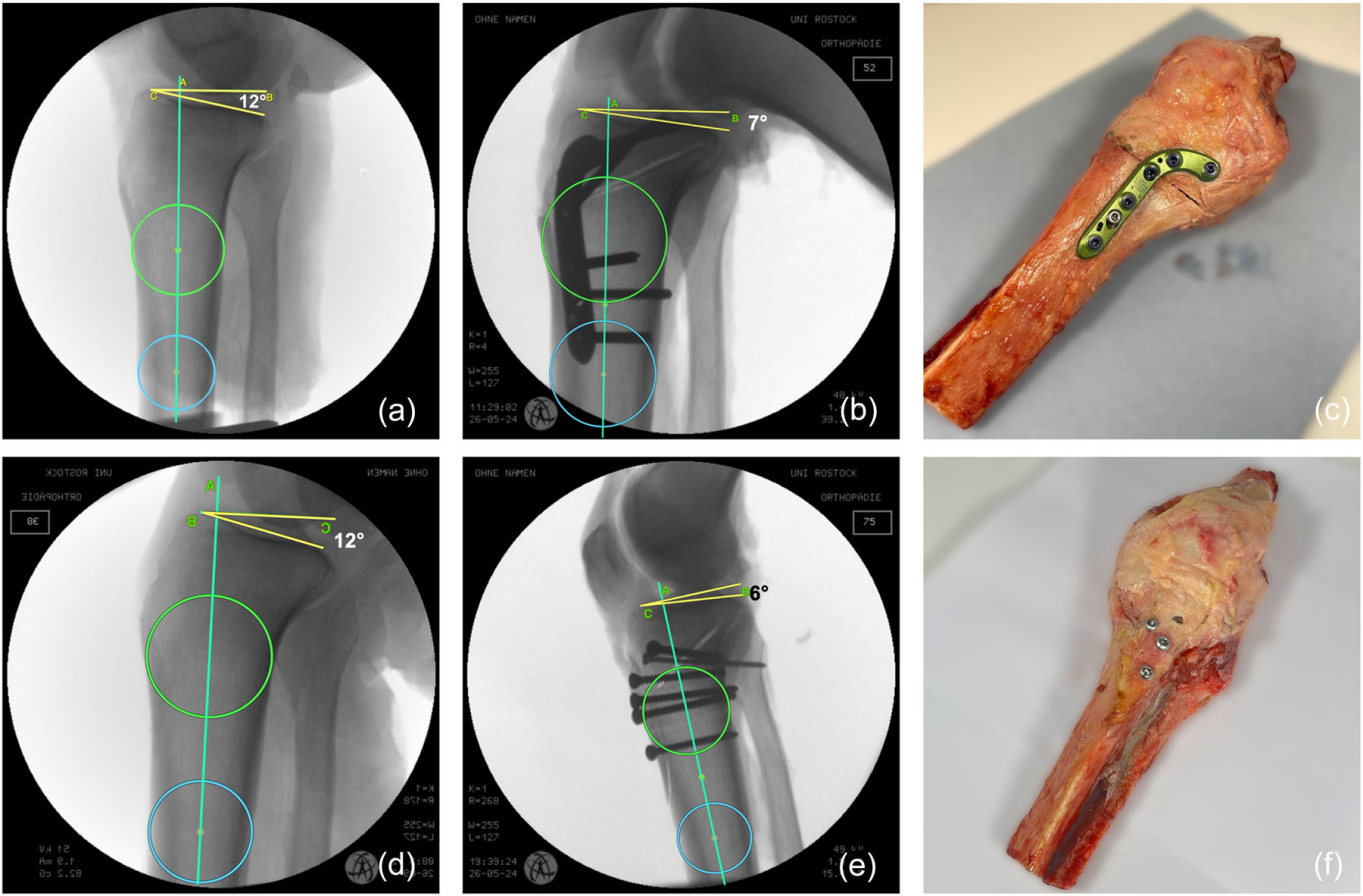
The lateral radiograph (a, d) demonstrated the measurement of PTS, which was measured using the diaphyseal axis, which was characterised by two circles that fit closely to the cortex of the tibial bone. The first circle was placed distal to the tibial tuberosity and the second circle was placed as far distally as the radiograph allowed [5]. Radiographs before (a, d) and after PTS correction (b, e) and cadaveric knee (c, f) after ACW‐HTO with infra‐tuberosity osteotomy stabilised with a standard osteotomy plate (b, c) and trans‐tuberosity technique with the tibial tuberosity as a “bioplate” (e, f). ACW‐HTO, anterior closed wedge high tibial osteotomy; PTS, posterior tibial slope.

For this study, 18 fresh‐frozen knee specimens from human cadavers, donated to the University Anatomical Program, were used with approval of the local ethics committee of the Rostock University Medical Center. Specimens with a history of previous knee surgery were excluded. No exclusion criteria regarding donor age or degenerative changes were applied. The specimens were stored at −20°C and thawed for 24 h prior to testing. They were transected above and below the knee joint and dissected down to the joint capsule using suitable surgical instruments. The patellar tendon and the quadriceps tendon remained intact as well as the joint capsule and the ligaments. The specimens were allocated in a paired donor‐matched manner into two intervention groups (angle‐stable plate and “bioplating” group), with one knee from each donor assigned to each intervention group. Side allocation was alternated between techniques, resulting in an equal distribution of left and right knees in both groups. PTS was measured before and after intervention on single‐shot lateral radiographs acquired using a mobile C‐arm, using the method described by Dejour et al. [5] (Figure 1). All PTS measurements were performed by a single observer. The bone density of the knee specimens was measured using dual‐energy X‐ray absorptiometry (DXA) and expressed as areal bone mineral density (g/cm^2^).

The intended PTS correction was set at 6° and verified using single‐shot lateral radiographs acquired with a mobile C‐arm. The intervention was performed with ACW‐HTO in both intervention groups using two common techniques under radiographic control by an experienced surgeon (Figure 1). In the case of the trans‐tuberosity technique described by Akoto et al. [2], an osteotomy of the tibial tubercle over a length of 4–6 cm was performed. After detachment of the tibial tubercle, two K‐wires at a 6°‐angle, pointing toward the tibial insertion of the posterior cruciate ligament (PCL) on the lateral radiograph were set indicating the plane of the PTS osteotomy. No dedicated fixed‐angle cutting guide was used. After completing the main osteotomy using an oscillating saw along the K‐wires, the bone wedge was removed and the osteotomy was carefully closed anteriorly. The fixation of the slope and the tibial tubercle osteotomy was achieved by using the tibial tubercle as a “bioplate”. Six 3.5 mm bicortical lag screws in an anterior to posterior direction through the tibial tubercle and the dorsal cortical bone of the tibia stabilised both osteotomies. Six screws were selected within the previously described range of three to eight lag screws for this technique [2].

The second technique was an infra‐tuberosity osteotomy as described by Hees et al. [9], where the PTS correction osteotomy started distal to the insertion of the patellar tendon. At first, two K‐wires were set determining the level of the osteotomy in the same manner as in the previously described technique. The procedure of sawing and closing the osteotomy was identical to the “bioplating” technique. Only the fixation technique differed—for this purpose, a standard osteotomy plate (“NewClip” Active motion Deflexion Osteotomy Plate, Newclip Technics, Haute Goulaine, France) was placed on the anteromedial tibial head, where two angle‐stable screws fixed the proximal part of the osteotomy. Then, the osteotomy site was closed by a compression screw in the distal fragment. The fixation was completed by angle‐stable screws resulting in an osteosynthesis with an angle‐stable anteromedial plate fixed by three angle‐stable screws in the proximal fragment and two angle‐stable and one compression screw in the distal fragment. In all cases the osteotomy was closed completely, and the dorsal tibial hinge was left intact, which was controlled and documented by image intensifier.

### Biomechanical testing

**Figure 2:**
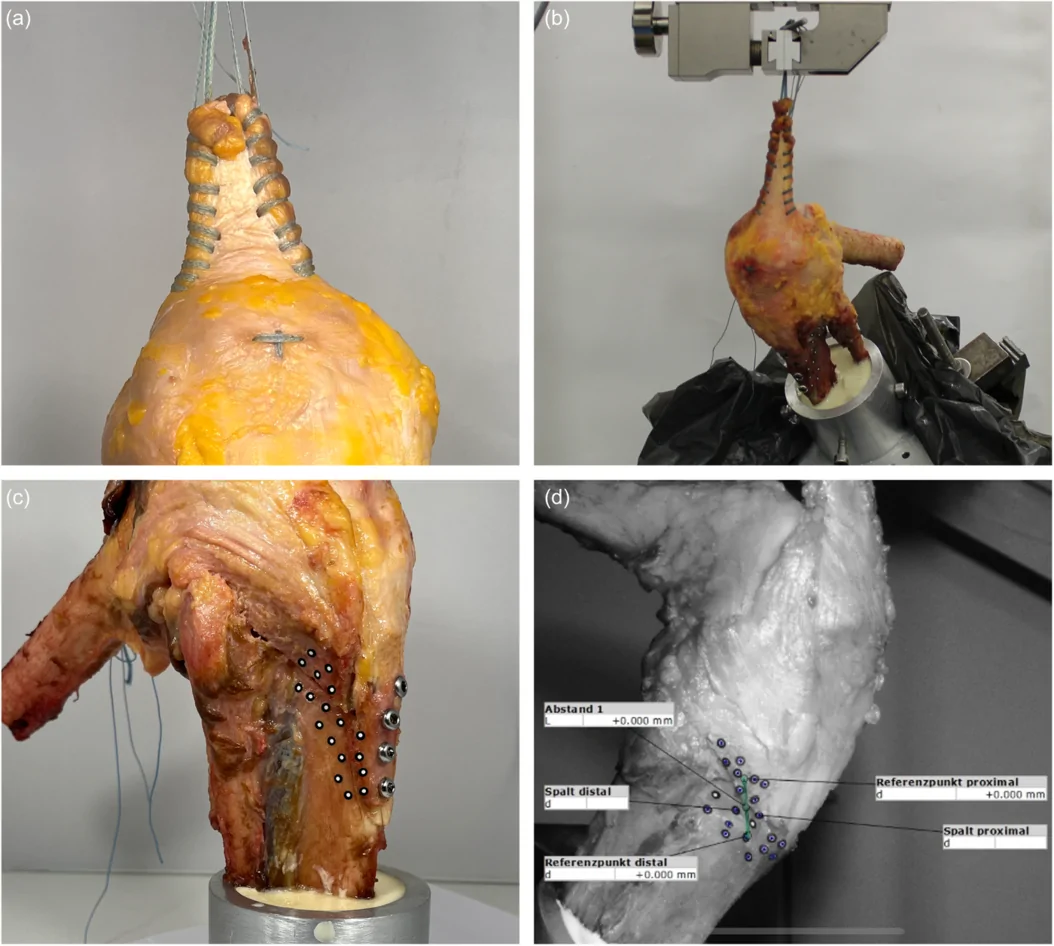
Experimental setup. Quadriceps tendon of the knee joint was fixed with the double baseball suture with a FiberTape® (Arthrex, Inc., Naples, FL, USA) (a) and placed in an electrodynamic testing machine at a flexion angle of 45° (b). The micromovements were recorded using a 3D optical measurement system with point markers placed on the tibia around the osteotomy gap (c) and analysed using the software GOM Suite (d).

For further biomechanical investigations, the prepared bone samples were embedded proximally and distally in resin (Goeßl & Pfaff, Karlskron, Germany). The biomechanical evaluation was conducted in two consecutive tests, i.e. cyclic loading followed by load‐to‐failure, using an electrodynamic test machine (LTM 5, ZwickRoell GmbH & Co. KG, Ulm, Germany). For this, the bones were positioned at a flexion angle of 45° in the test machine and the tensile load was applied via a “baseball” suture with a FiberTape® (Arthrex, Inc., Naples, FL, USA) attached to the quadriceps femoris tendon (Figure 2a). A flexion angle of 45° was chosen because previous biomechanical testing demonstrated maximum patellar and quadriceps tendon forces between 30° and 60° of knee flexion [10]. The specimens were preloaded with 5 N, and 1000 sinusoidal tensile loading cycles between 50 N and 200 N were subsequently applied at 1 Hz under force control. Following the cyclic loading, the specimens were loaded to failure at a rate of 10 mm/s under position control (Figure 2b). The force values were measured continuously until failure of the system. Specifically, the force at first significant mechanical collapse, defined as the first significant drop in mechanical resistance accompanied by visible displacement in the optical analysis, was interpreted as osteotomy failure. The maximum measured force was also evaluated. Six additional intact knee specimens underwent identical load‐to‐failure testing to verify that the quadriceps tendon–suture loading construct was not the limiting component of the experimental setup. These specimens were not included in the figures and analysis.

### Optical evaluation of micromovements

The micromovements between the distal and proximal parts of the tibial osteotomy, resulting from tensile loading of the patellar tendon, were recorded using an optical 3D measurement system (ARAMIS 5 M, 23 mm lenses, Carl Zeiss GOM Metrology GmbH, Braunschweig, Germany) and analysed in GOM Suite (v3.1, Carl Zeiss GOM Metrology GmbH, Braunschweig, Germany) (Figure 2c). For this purpose, point markers were equally placed on the tibial bone around the osteotomy gap. During cyclic loading, 10 cycles were recorded at 15 Hz starting at cycles 200 and 800, respectively. Furthermore, the micromovement during pull‐to‐failure was recorded with 5 Hz starting from the beginning of the test. For the GOM ARAMIS system, an accuracy of 0.001 mm has been reported in the literature [19, 22].

In GOM Suite, reference points distal and proximal to the osteotomy gap were defined using all continuously recorded points. The relative change in the distance between these reference points was evaluated, corresponding to the gap opening under tensile loading of the quadriceps tendon.

### Statistical analysis

Data were collected and summarised using Microsoft Excel. Statistical analyses were performed using GraphPad Prism 10 (GraphPad Software, Inc, San Diego, California, USA). A formal a priori power analysis was not performed due to the limited availability of human cadaveric specimens. Instead, a sample representing the largest feasible cohort was utilised to maximise the potential to detect biomechanical effects. Two‐way analysis of variance (ANOVA) and Bonferroni multiple comparison tests were used for significance testing. Values of *p* < 0.05 were considered statistically significant.

## RESULTS

Twelve knee specimens (*n* = 6 per intervention group) with a mean donor age of 79.2 ± 8.2 years were included in the comparative analysis. The PTS measurements, age of the donors and group distribution are shown in Table 1.

**Table 1:** Composition of the knee specimens in both intervention groups regarding sex, side, age, bone density and pre‐ and post‐intervention PTS distribution.

	Number	Sex (w:m)	Side (r:l)	Age (years)	BMD (g/cm^2^)	PTS pre	PTS post	∆ PTS
Plate	*n* = 6	1:2	1:1	79.2 ± 8.2	1.2 ± 0.2	10° ± 2.8°	4.8° ± 2.8°	5.2° ± 2.6°
Bioplate	*n* = 6	1:2	1:1	79.2 ± 8.2	1.1 ± 0.1	10.1° ± 2.3°	4.4° ± 1.1°	5.8° ± 1.5°

Abbreviations: BMD, bone mineral density; PTS, posterior tibial slope.

### Biomechanical testing

**Figure 3:**
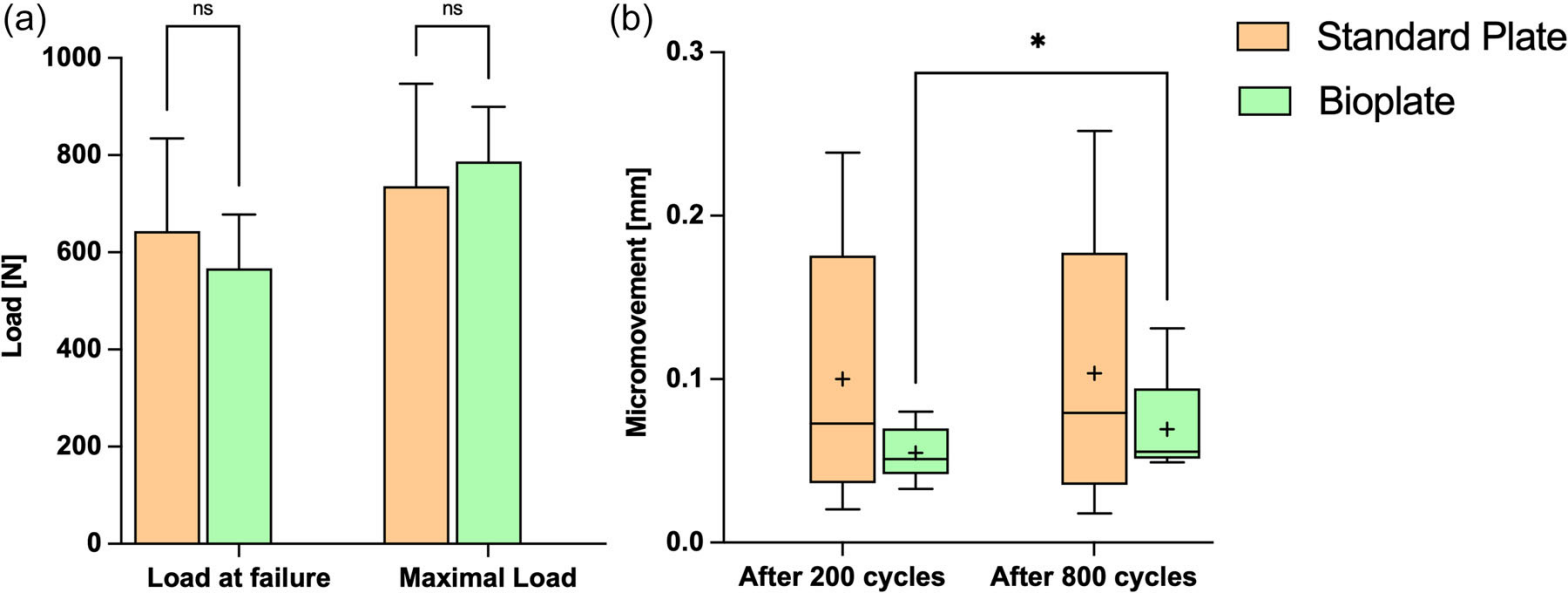
Results of tensile and micromovement testing: (a) The bar chart (mean with SD (standard deviation)) shows the measured load capacity at the first failure of the osteotomy and the maximum measured load (in Newtons). (b) The boxplots (min to max) show the micromovement of the osteotomy gap in mm (y‐axis), recorded for 10 cycles at 15 Hz, starting at cycles 200 and 800 (x‐axis). Statistical analysis was performed with a two‐way ANOVA and Bonferroni post‐hoc test. All significant differences (*p* < 0.05) were indicated by *. ANOVA, analysis of variance; ns, not significant; SD, standard deviation.

There was no significant difference in measured load capacity between the two osteotomy techniques, either at first failure or for maximum measured load (Figure 3a). The mean force measured at the time of the first failure of osteosynthesis was 643.9 ± 190.8 N in the angle‐stable plate group and 566.8 ± 111.0 N in the “bioplating” group (*p* = 0.422). There were also no significant differences between the two intervention groups in terms of maximum recorded tensile load values (736.0 ± 211.4 N vs. 786.9 ± 112.4 N, *p* = 0.594). In all knee specimens of the intervention groups, failure of the osteosynthesis led to termination of the test. In the six additional intact specimens used for setup validation, failure occurred by suture disruption at 1386.8 ± 215.4 N, substantially above the mean osteosynthesis failure loads observed in either intervention group.

### Optical evaluation

After 200 loading cycles, no significant difference in micromovement at the osteotomy gap was found between the two intervention groups (0.100 ± 0.083 mm vs. 0.055 ± 0.017 mm, *p* = 0.515). Similarly, there was no significant difference between the two groups after 800 cycles (0.104 ± 0.087 mm vs. 0.069 ± 0.035 mm, *p* = 0.889). However, micromovement in the “bioplating” group was significantly increased after 800 cycles compared to 200 cycles (0.055 ± 0.017 mm vs. 0.069 ± 0.035 mm, *p* = 0.025). Such an increase in micromovement was not observed between 200 cycles and 800 cycles in the group with plate osteosynthesis (0.100 ± 0.083 mm vs. 0.104 ± 0.087 mm, *p* = 0.518) (Figure 3b).

## DISCUSSION

The main finding of this experimental study was that fixation using either an angle‐stable plate or the ‘bioplating’ method after ACW‐HTO for PTS correction showed similar resistance to tensile load applied through the quadriceps tendon and could therefore be considered similarly stable. Nevertheless, increased micromovement of the osteotomy gap was observed in the “bioplating” group, while the micromovement in the angle‐stable group remained almost constant with increasing cycles.

The ACW‐HTO is considered a relatively stable osteotomy as the rate for delayed bone union was 0.6% in a cohort of 170 ACW‐HTO cases [24]. Due to the triangular shape of the proximal tibia and the oblique osteotomy cut there is a large bone interface and a wide dorsal tibial hinge. Compared to other osteotomies around the knee the risk for relevant hinge fractures is low, in the aforementioned study of 170 ACW‐HTOs no hinge fracture could be observed [8, 15, 16, 24]. Because ACW‐HTO is considered stable under axial load, tensile forces of the quadriceps tendon were evaluated, as they may be a decisive factor during gait and may lead to early failure of the osteosynthesis.

The load‐to‐failure test demonstrated relevant findings regarding osteosynthesis failure. In all cases, the failure occurred at the osteotomy site due to applied tensile forces, accompanied by dislocation of some screws. The additional load‐to‐failure testing of intact specimens demonstrated that neither the sutures nor the quadriceps tendon preparation were the limiting factors, but rather the osteosynthesis of the osteotomy site. Several previous studies reported different patellofemoral joint forces depending on activities of daily living. However, the measurements of the patellofemoral joint reaction force are not directly comparable with the results of the present study, as the loading forces in the present setup were exerted on the patellar and quadriceps tendons. The literature reports maximum patellar tendon forces of 5452.7 N for adult men and 2168.8 N for young women measured in an in vitro setup [17]. Osteotomies in the present study failed at a load of 644 N (angle‐stable plate) and 567 N (“bioplating”) respectively, without statistically significant difference. However, the absence of statistically significant differences may be related to the small sample size and the limited statistical power to detect true differences. Therefore, these findings should be interpreted with caution and must not be regarded as evidence of non‐inferiority.

In the present biomechanical setup, a flexion angle of 45° was chosen, as prior biomechanical tests revealed maximum forces to the patellar tendon and the quadriceps tendon at flexion angles between 30° and 60° compared to 90° and 120° [10]. The same study showed that, depending on the flexion angle of the knee, tensile forces of up to 470 ± 118 N on the patellar tendon and 532 ± 155 N on the quadriceps tendon can occur during gait [10]. Furthermore, peak values of approximately 600 N for the patellar tendon and around 900 N for the quadriceps tendon during cycling have been reported in the literature [21]. These values are highly relevant considering the findings of the present study, as, considering only the experimental results, normal gait alone could potentially lead to failure of the osteotomy. When synthesising the loads associated with activities of daily living with the present results, protocols allowing full weight‐bearing at flexion angles above 30° may be viewed with caution, since peak forces at the osteotomy site typically occur around 60° of knee flexion. An important aspect is that the specimens were tested with an intact proximal tibia and without pre‐existing or concomitantly created ACL tibial tunnels. As ACW‐HTO for PTS reduction is mostly performed in the setting of revision ACL reconstruction, the present findings may not fully reflect fixation behaviour in tunnel‐compromised bone stock. On the other hand, several other substantial limitations must be acknowledged before drawing conclusions regarding the postoperative treatment regimen. Factors such as the time‐zero model, the elderly patient cohort, the fixed flexion angle, and the lack of axial loading significantly influence the translation of present in vitro failure loads to in vivo conditions. Consequently, these variables must be carefully weighed before adapting postoperative protocols, such as weight‐bearing or ROM restrictions.

Importantly, although no significant between‐group difference in micromovement was observed, the “bioplating” group demonstrated a significant increase in micromovement from 200 to 800 loading cycles. However, as the magnitude of this motion was minimal and literature defining clinically relevant thresholds is lacking, any recommendations for postoperative treatment derived from the findings of this study must be interpreted in light of the limitations of the biomechanical setup. Those are detailed in the following section.

### Limitations

This experimental study has several limitations. For instance, the complex forces acting on the knee joint during gait, which can influence bone healing after osteotomy, cannot be fully reproduced in this biomechanical setup. Future studies should therefore investigate a range of knee flexion angles using a knee simulator [27]. In the present study, the forces considered most relevant for bone healing and fixation stability in this specific osteotomy were reproduced. Moreover, this biomechanical study only assessed primary stability, as it represents a time‐zero experimental setup. Secondary stability, which develops through bone healing and is influenced by numerous biological and mechanical factors, cannot be evaluated under these conditions. Furthermore, the present findings have to be interpreted as applying primarily to intact proximal tibiae without ACL tunnels. The findings of this study therefore need to be confirmed in further biomechanical and clinical investigations. Additionally, the donors of the specimens were older than patients undergoing PTS osteotomy. The paired donor‐matched allocation ensured an identical donor age distribution between the two intervention groups. Nevertheless, degenerative changes were not used as an exclusion criterion and may therefore have influenced the absolute biomechanical measurements. Consequently, the maximum tolerated load in younger patients with higher bone density after ACW‐HTO may exceed the values measured in this biomechanical study. Furthermore, the intended 6° correction was based on radiograph K‐wire positioning without a dedicated fixed‐angle cutting guide, which may have introduced variability in the achieved PTS correction. PTS measurements were performed by a single observer without repeated measurements; therefore, study‐specific intra‐ and interrater reliability could not be assessed.

## CONCLUSION

The maximal load at failure of the osteosynthesis did not differ significantly between the two surgical techniques. Nevertheless, micromovement increased significantly from 200 to 800 loading cycles in the “bioplating” group, whereas no significant increase was observed with angle‐stable plate fixation. Transferring these findings to postoperative treatment after ACW‐HTO suggests that early weight‐bearing during level walking or cycling should be carefully considered at flexion angles greater than 30°; however, the clinical transferability of the present measurements is limited by the time‐zero design, the elderly cohort, and the absence of axial loading, all of which must be addressed in further biomechanical studies before definitive treatment recommendations can be established.

## AUTHOR CONTRIBUTIONS

Andrzej Jasina, Christoph Lutter, Felix Ferner, Pia Metje, Jan‐Oliver Sass and Rainer Bader conceived and designed the study. Data collection and extraction were performed by Andrzej Jasina, Pia Metje, Jan‐Oliver Sass, Jessica Hembus, Sjard Simons, Parisa Pourostad, Felix Ferner, and Christoph Lutter. Data analysis and interpretation were carried out by Andrzej Jasina, Maeruan Kebbach, Sina Gräber, Kira Liebau, Parisa Pourostad, Christoph Lutter, Felix Ferner, Rainer Bader, and Philipp Schuster. Andrzej Jasina drafted the manuscript with substantial contributions from Felix Ferner and Christoph Lutter. All authors critically revised the manuscript for important intellectual content and approved the final version.

## FUNDING INFORMATION

The authors have nothing to report.

## CONFLICT OF INTEREST STATEMENT

Prof. Philipp Schuster received speaker fees and receives royalties from Newclip Technics (Haute Goulaine, France). All other authors declare no conflict of interest.

## ETHICS STATEMENT

Ethical approval for this study was obtained from the local ethics committee of Rostock University Medical Center (Rostock, Germany), reference number A 2020‐0098.

## Data Availability

The data that support the findings of this study are available from the corresponding author upon reasonable request.
